# Feature instructions improve face-matching accuracy

**DOI:** 10.1371/journal.pone.0193455

**Published:** 2018-03-15

**Authors:** Ahmed M. Megreya, Markus Bindemann

**Affiliations:** 1 Department of Psychological Sciences, College of Education, Qatar University, Doha, Qatar; 2 School of Psychology, Keynes College, University of Kent, Canterbury, United Kingdom; Bournemouth University, UNITED KINGDOM

## Abstract

Identity comparisons of photographs of unfamiliar faces are prone to error but important for applied settings, such as person identification at passport control. Finding techniques to improve face-matching accuracy is therefore an important contemporary research topic. This study investigated whether matching accuracy can be improved by instruction to attend to specific facial features. Experiment 1 showed that instruction to attend to the eyebrows enhanced matching accuracy for optimized same-day same-race face pairs but not for other-race faces. By contrast, accuracy was unaffected by instruction to attend to the eyes, and declined with instruction to attend to ears. Experiment 2 replicated the eyebrow-instruction improvement with a different set of same-race faces, comprising both optimized same-day and more challenging different-day face pairs. These findings suggest that instruction to attend to specific features can enhance face-matching accuracy, but feature selection is crucial and generalization across face sets may be limited.

## Introduction

The comparison of one face photograph to another, to establish whether these depict the same person, is surprisingly difficult when the depicted targets are not known to the observer (for reviews, see, e.g., [1–4]). This difficulty is observed when identity comparisons are based on highly-similar same-day photographs of a person [5–7], or when these require the matching of a face photograph to a live person [8–10]. This suggests a person identification problem that is already present under seemingly simple and highly favourable task conditions. Identification accuracy declines further under more challenging conditions, such as when to-be-compared images are taken months apart [11], or when identification requires comparison of strictly controlled high-quality face portraits with unconstrained ambient images of faces [12,13].

The documented difficulty of this task has important implications, as this type of face matching provides one of the primary means for person identification at airports and national borders. Research indicates that passport and security officers in these settings are also prone to making identification errors [10,14]. However, it is now also becoming clear that professionals working in these settings can vary in their facial identification accuracy. For example, the performance of individual passport officers in an optimized face-matching test ranges from near-chance to near-perfect, displaying a very broad range in individual ability [10]. Moreover, these differences are seemingly unrelated to experience, suggesting that the prolonged practice of face matching is not sufficient by itself to improve this ability.

The poor performance of some passport officers [10,14], and the importance of face-matching procedures in real-life security settings [15], demonstrate a critical need for finding techniques that can improve performance in this task. Several possibilities are currently under investigation, such as the administration of performance feedback [16,17], exposure to within-identity variation [13,18–20], aggregation of different individuals’ decisions [21,22], and redesign of photo-ID to include multiple or averaged facial images [18,20]. However, these methods have limitations that may make their implementation in applied settings challenging. For example, whereas trial-by-trial feedback can enhance face-matching performance [16,17], this is difficult to implement outside of the laboratory, where the accuracy of decisions is not known. Similarly, whereas aggregation of responses can produce stark improvements in accuracy [22], this method is not applicable in settings where identification decisions for a given target person are made by only a single individual, or where data-sharing for response aggregation is not easily possible.

In this study, we therefore investigate an alternative method for improving face-matching accuracy. Our approach is based on directing observers through task instructions to pay attention to particular facial features to determine whether this can confer a benefit in performance. This simple approach is worthy of investigation for two reasons. Firstly, it is now well established that the provision of only one or two images of a person’s face, as is the case in face matching, provides very limited information about the *general* appearance of that person [3,23,24]. Such limited instances do not adequately capture the many ways in which a person’s appearance can vary naturally. To compensate, unfamiliar face matching must inevitably place greater emphasis on the face *image* at hand rather than the depicted face *identity*. Consequently, unfamiliar face matching is held to rely on basic pictorial, or image-based, information [2,7,11,25,26]. Indeed, unfamiliar face matching is not associated with the highly accurate process of *familiar* face recognition [7] or a strong reliance on the processing of faces as holistic gestalts, which is typical of familiar face recognition [27]. Instead, unfamiliar face matching correlates with tasks that require the feature-based processing of non-face objects [6,7]. This indicates that face matching is more of a feature-based process that relies on comparison of individual facial landmarks, such as the eyes, nose or mouth. If that is the case, then it should be possible to improve matching accuracy by directing observers to features that are particularly beneficial for such identification.

The second reason for investigating whether face-matching accuracy can be improved with a feature-based approach is that feature-by-feature comparison training is already commonly offered to professionals in relevant occupations, such as passport officers (for reviews, see, e.g., [15,28]). However, limited data are available as to whether such approaches actually improve face-matching performance. A recent study investigated this directly by asking observers to rate the similarity of features of two faces before making identity-match versus mismatch decisions [29]. Eleven different facial features were rated, ranging from external features (such as the ears, face shape, and jawline) to internal features (such as the eyes, nose, and mouth). Rating the similarity of these features improved matching accuracy on identity match trials, but not on mismatch trials. Moreover, this improvement was not observed when similarity ratings were based on the perceived personality traits of faces, which is held to engage holistic processing (for a review, see, e.g., [30]). Towler et al. [29] also assessed a group of professional forensic facial examiners, who demonstrated superior face-matching accuracy compared to untrained student participants, whilst also producing feature similarity ratings that were more diagnostic of facial identity. Taken together, these findings suggest that feature-based approaches can enhance face-matching accuracy, though such gains may be limited to identity matches.

In this study, we seek to investigate feature-based strategies further, by assessing whether a similar advantage can be observed with a more direct approach. In contrast to Towler et al. [29], who required observers to respond to the full set of eleven individual features before making an identification, we simply asked observer to pay particular attention to a key feature. We then assessed whether these instructions were sufficient for generating a performance gain, and whether instructions for some features improved accuracy more than for others. We specifically focused on two main features that have been linked to person identification in previous work, comprising the eyes [31] and eyebrows [32]. In addition, we focused on the ears, which appear to be particularly useful for biometric identification [33], and also appear to relate strongly to accuracy in unfamiliar face matching [29].

As an additional aim, we sought to investigate whether any benefit of feature instructions for improving matching accuracy would be limited to faces of an observer’s own race or generalize to those of another race, by comparing Arab observers’ matching of Arab and Caucasian faces. Whilst this is an important issue for applied settings, in which professionals have to process people from a range of ethnic backgrounds, it is difficult to predict whether own- and other-race faces would benefit differentially from feature instructions. On one hand, face recognition and face matching is consistently more challenging for faces of a race other than one’s own (see, e.g., [34–36]). This could suggest that the matching of other-race faces might be enhanced to a greater extent by feature instructions, as accuracy could be improved more substantially for these stimuli. On the other hand, a number of studies indicate also that the processing of other-race faces is comparatively more dependent on features than that of same-race faces [37–40]. Thus, there might also be less scope for other-race face-matching to improve with feature-based instructions.

In addition, this study also aimed to explore which facial features (the eyebrows, eyes, or ears) are beneficial for improving the matching of own- and other-race stimuli. Faces of different races can differ in the extent to which specific facial features carry identity information, and instruction to focus on race-specific individuating features can help to increase recognition accuracy. For example, in face memory tasks, initial fixations on the eyes increase recognition accuracy for White faces irrespective of observer race, whereas accuracy for Black faces is enhanced by fixations on the nose [41–42]. It is possible that a similar effect is observed here with face matching, with different feature conditions enhancing accuracy for same- and other-race faces. However, it is difficult to predict which features might be beneficial for same- and other-race faces. Evidence on the diagnosticity of individual features for face matching is limited and mixed with regard to the features and races under investigation here. Abudarham and Yovel [43], for example, found that observers possessed greater perceptual sensitivity for information carried by eye shape than ear protrusion, but not for eye size, eyebrow shape, or eye distances. Moreover, these data were obtained with Caucasian faces and participants at a Mediterranean university. By contrast, Towler et al. [29] found that similarity ratings of ears provide better diagnosticity for discriminating identity matches and mismatches than of eyes, but did not test the eyebrow regions or for race effects. The available evidence therefore makes it difficult to predict which feature instructions might be most useful for improving matching of same- and other-race faces in the current study.

## Experiment 1

This experiment examined whether face-matching accuracy can be improved by giving instruction to attend to specific facial features, and assessed whether some feature instructions confer a greater improvement than others. Observers completed an initial block to provide a baseline measure of their face-matching accuracy. This was followed by specific task instructions to focus on the eyes, eyebrows or ears, to assess the benefit of these features on a between-subject basis. The effect of these instructions on face-matching accuracy was then assessed with a second block of trials. In addition, we assessed the impact of feature instructions with same-race faces (Arab) and other-race faces (Caucasian), to determine if any improvements in performance generalize across race.

### Method

#### Participants

Sixty under-graduate students from Qatar University volunteered to participate in this experiment (*M*_age_ = 21.1, *SD*_age_ = 0.9; 70% females). All reported normal or corrected to normal vision. Ethical approval for participation in this study was provided by Qatar University’s institutional review board (QU-IRB) and all methods were administered in accordance with QU-IRB guidelines and regulations.

#### Stimuli

A total of 240 face pairs were employed as stimuli in this experiment. These comprised 120 Arab face pairs, of 60 identity matches and 60 identity mismatches, and corresponding numbers of Caucasian face pairs. These stimuli were constructed from 90 Arab and 90 Caucasian identities, so that none of the identities occurred in more than one match pair and two mismatches. The Arab face pairs were taken from an Egyptian face-matching database [9], whilst the Caucasian pairs were derived from the UK Home Office PITO database [7,44]. Each pair consisted of a video still image of a face and a digital face photograph, which either depicted the same person (identity matches) or two different people (identity mismatches). For each person, the video stills and digital photographs were taken only moments apart and under the same lighting conditions. In addition, all faces were shown in a frontal view, with a neutral expression, without extraneous background, and in greyscale. Note that all faces were male, as suitable face photographs of Arab females were unavailable due to the headscarf culture. The size of each face image was approximately 5 x 7 cm. Examples of Arab and Caucasian face pairs can be found in [9,25].

#### Procedure

Participants were tested individually in a laboratory on an Apple Macintosh laptop running Superlab Pro software, which was used to present stimuli and record participants’ responses. Each participant performed two blocks of 60 face pairs, comprising 15 Arab identity matches and 15 mismatches, and 15 Caucasian matches and 15 mismatches. These stimuli were displayed until a response was registered, and participants were asked to classify these as identity matches and mismatches as accurately as possible by pressing one of two labeled buttons on the computer keyboard. In between both blocks, observers were given feature comparison instructions. These emphasized either the importance of the eyebrows, eyes or ears for face matching accuracy, and were administered on a between-subject basis (with N = 20 per group). Translated from Arabic, the instructions stated “*Now you are going to perform the same task but please focus on the eyebrows/eyes/ears*. *Compare this feature between the images and accordingly make your same or different identity decision*”. Different face pairs were employed in each block and presented in a unique random order for each participant. However, the presentation of each target face in an identity match or mismatch, and the presentation of all face pairs across blocks and conditions were counterbalanced across observers over the course of the experiment.

### Results

#### Matching of same-race faces

**Fig 1:**
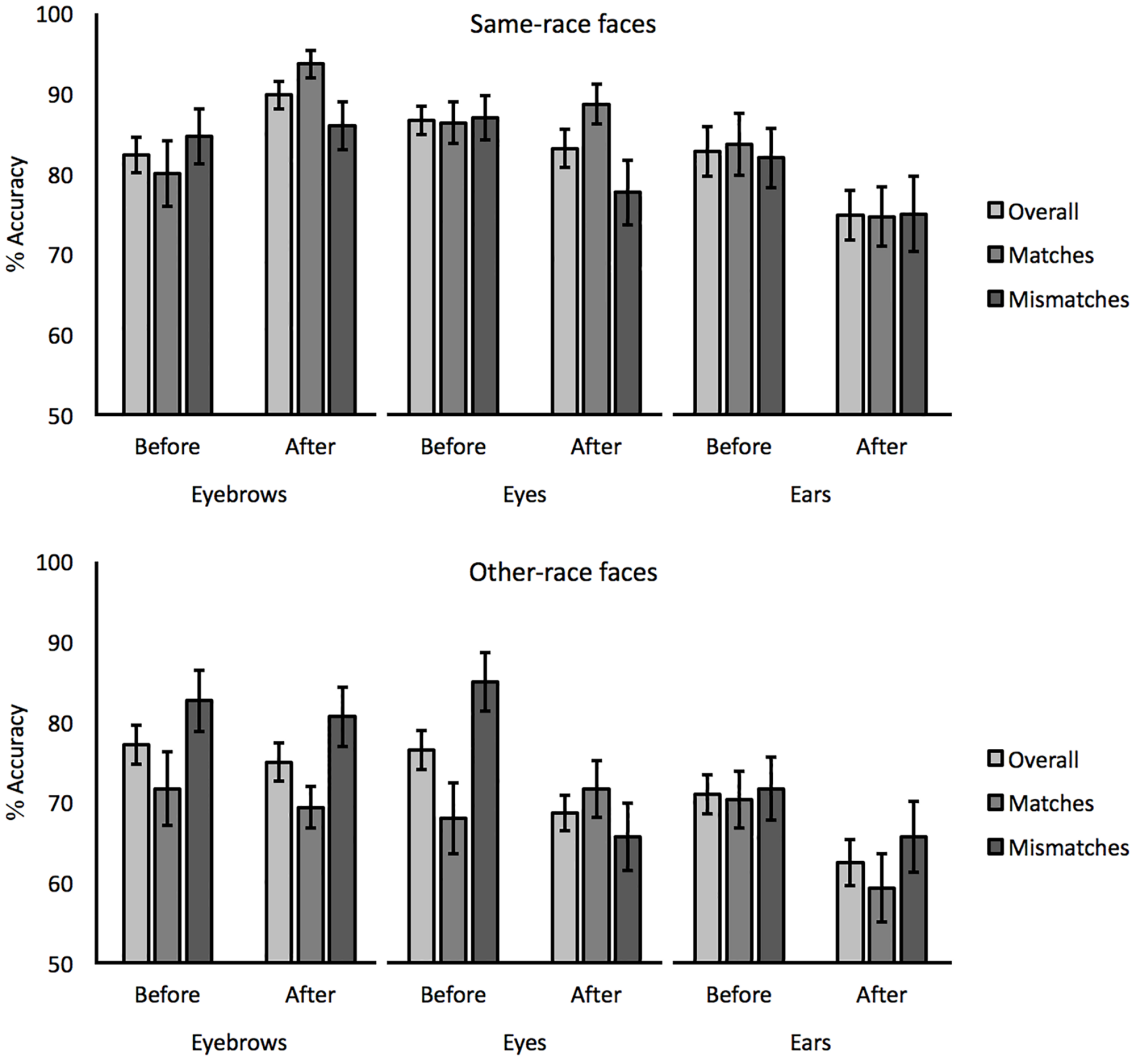
Overall, match and mismatch accuracy for same-race faces and other-race faces before and after the administration of eyebrows, eyes and ears feature instructions in Experiment 1. Error bars reflect standard errors of the means.

For same-race (Arab) faces, performance was analyzed in terms of the percentage accuracy of correct match and mismatch responses, as well as overall accuracy (i.e., the mean of the two). These data are illustrated in Fig 1. A 3 (feature: eyebrows, eyes, ears) x 2 (instruction: before vs. after) mixed-factor ANOVA of overall accuracy did not find a main effect of instruction, *F*(1,57) = 1.05, *p* = 0.31, *ƞ*_*p*_^*2*^ = 0.02, but a main effect of feature, *F*(2,57) = 3.21, *p* < 0.05, *ƞ*_*p*_^*2*^ = 0.10, and an interaction between these factors, *F*(2,57) = 12.56, *p* < 0.001, *ƞ*_*p*_^*2*^ = 0.31. A series of paired-sample *t*-tests (with alpha adjusted to 0.05/3 = 0.017 for three comparisons) revealed an improvement after instruction in the eyebrows condition (82.3% vs. 89.9%), *t*(19) = 4.79, *p* < 0.001, *d* = 0.84, no difference in accuracy in the eyes condition (86.7% vs. 83.2%), *t*(19) = 1.48, *p* = 0.16, *d* = 0.39, and a decline in performance in the ears condition (82.8% vs. 74.8%), *t*(19) = 2.99, *p* < 0.017, *d* = 0.57.

A 3 (feature) x 2 (instruction) ANOVA of match trials did not find a main effect of instruction, *F*(1,57) = 1.11, *p* = 0.30, *ƞ*_*p*_^*2*^ = 0.02. However, a marginally significant main effect of feature, *F*(2,57) = 3.15, *p* = 0.05, *ƞ*_*p*_^*2*^ = 0.10, and an interaction of feature and instruction was found, *F*(2,57) = 8.72, *p* < 0.001, *ƞ*_*p*_^*2*^ = 0.23. This was due to an improvement with instruction in the eyebrows condition (80.0% vs. 93.7%), *t*(19) = 3.60, *p* < 0.001, *d* = 0.99, but more comparable performance before and after instruction in the eyes condition (86.3% vs. 88.7%), *t*(19) = 0.69, *p* = 0.50, *d* = 0.23, and the ears condition (83.7% vs. 74.7%), *t*(19) = 2.10, *p* = 0.05, *d* = 0.50.

Finally, a 3 (feature) x 2 (instruction) ANOVA of mismatch trials revealed a main effect of instruction, *F*(1,57) = 5.99, *p* < 0.05, *ƞ*_*p*_^*2*^ = 0.10, due to generally lower accuracy after instruction (84.5% vs. 79.6%). The main effect of feature, *F*(2,57) = 2.51, *p* = 0.09, *ƞ*_*p*_^*2*^ = 0.08, and the interaction of feature and instruction, *F*(2,57) = 1.14, *p* = 0.33, *ƞ*_*p*_^*2*^ = 0.04, was not significant.

#### Matching of other-race faces

The percentage accuracy data for other-race (Caucasian) faces are also illustrated in Fig 1. A 3 (feature) x 2 (instruction) mixed-factor ANOVA of overall accuracy found a main effect of instruction, *F*(1,57) = 14.78, *p* < 0.001, *ƞ*_*p*_^*2*^ = 0.21, but due to higher accuracy before than after receiving feature instruction (74.9% vs. 69.7%). In addition, a main effect of feature was also found, *F*(2,57) = 5.35, *p* < 0.01, *ƞ*_*p*_^*2*^ = 0.16. A series of paired-sample *t*-tests (with alpha adjusted to 0.05/3 = 0.017 for three comparisons) showed that this was due to lower accuracy for ears (66.7%) versus eyebrows (76.1%), *t*(19) = 2.77, *p* < 0.017, *d* = 0.95. Accuracy did not differ between eyebrows (76.1%) and eyes (72.6%), *t*(19) = 1.16, *p* = 0.26, *d* = 0.40, or between eyes (72.6%) and ears (66.7%), *t*(19) = 1.96, *p* = 0.06, *d* = 0.64. The interaction of feature and instruction was not significant, *F*(2,57) = 1.57, *p* = 0.22, *ƞ*_*p*_^*2*^ = 0.05.

An analogous ANOVA of match accuracy did not show a main effect of instruction, *F*(1,57) = 1.23, *p* = 0.27, *ƞ*_*p*_^*2*^ = 0.02, of feature, *F*(2,57) = 1.09, *p* = 0.34, *ƞ*_*p*_^*2*^ = 0.04, or an interaction of these factors, *F*(2,57) = 2.14, *p* = 0.13, *ƞ*_*p*_^*2*^ = 0.07. However, for mismatch trials, an interaction of feature and instruction was found, *F*(2,57) = 7.61, *p* < 0.001, *ƞ*_*p*_^*2*^ = 0.21. A series of paired-sample *t*-tests (with alpha adjusted to 0.05/3 = 0.017 for three comparisons) revealed a decline in accuracy from before to after feature instructions in the eyes condition (85.0% vs. 65.7%), *t*(19) = 5.56, *p* < 0.001, *d* = 1.11, whereas before- and after-instructions accuracy for eyebrows (82.7% vs. 80.7%), *t*(19) = 0.69, *p* = 0.50, *d* = 0.12, and ears (71.7% vs. 65.7%), *t*(19) = 1.74, *p* = 0.10, *d* = 0.33, did not differ.

#### Sensitivity and bias for same-race faces

For completeness, the accuracy data were also transformed into signal detection measures of sensitivity (*d’*) and bias (*criterion*). For same-race (Arab) faces, a 3 (feature) x 2 (instruction) mixed-factor ANOVA of *d’* did not show a main effect of instruction, *F*(1,57) = 0.47, *p* = 0.50, *ƞ*_*p*_^*2*^ = 0.01, but revealed a main effect of feature, *F*(2,57) = 3.34, *p* < 0.05, *ƞ*_*p*_^*2*^ = 0.11, and an interaction between factors, *F*(2,57) = 8.68, *p* < 0.001, *ƞ*_*p*_^*2*^ = 0.23. A series of paired-sample *t*-tests (with alpha adjusted to 0.05/3 = 0.017 for three comparisons) revealed an increase in sensitivity after receiving instructions in the eyebrows condition (*d’* before = 2.37, SD = 0.93 vs. *d’* after = 3.05, SD = 0.93), *t*(19) = 3.96, *p* < 0.001, *d* = 0.73, no difference in performance between before and after instruction in the eyes condition (*d’* before = 2.63, SD = 0.84 vs. *d’* after = 2.41, SD = 1.09), *t*(19) = 0.80, *p* = 0.43, *d* = 0.23, and a decline in accuracy after instruction in the ears condition (*d’* before = 2.39, SD = 1.28 vs. *d’* after = 1.64, SD = 1.10), *t*(19) = 2.77, *p* < 0.016, *d* = 0.63.

A 3 (feature) x 2 (instruction) mixed-factor ANOVA of *criterion* revealed a main effect of instruction, *F*(2,57) = 6.28 *p* < 0.05, *ƞ*_*p*_^*2*^ = 0.10, due to a bias to make more match decisions after than before instructions (*criterion* before = 0.04, SD = 0.31 vs. *criterion* after = -0.15, SD = 0.24). The main effect of feature, *F*(2,57) = 0.21, *p* = 0.82, *ƞ*_*p*_^*2*^ = 0.01, and the interaction of feature and instruction was not significant, *F*(2,57) = 2.34, p = 0.11, *ƞ*_*p*_^*2*^ = 0.08.

#### Sensitivity and bias for other-race faces

For other-race (Caucasian) faces, a 3 (feature) x 2 (instruction) mixed-factor ANOVA of *d’* found a main effect of instruction, *F*(1,57) = 22.08, *p* < 0.001, *ƞ*_*p*_^*2*^ = 0.28, but due to higher sensitivity before than after instructions (*d’* before = 1.75, SD = 0.49 vs. *d’* after = 1.17, SD = 0.40), *t*(19) = 4.49, *p* < 0.001, *d* = 1.30. In addition, a main effect of feature was also found, *F*(2,57) = 7.02, *p* < 0.01, *ƞ*_*p*_^*2*^ = 0.20. A series of paired-sample *t*-tests (with alpha adjusted to 0.05/3 = 0.017 for three comparisons) showed that this reflects lower sensitivity for ears than eyebrows (*d’* eyebrows = 1.86, SD = 0.86 vs. *d’* ears = 1.01, SD = 0.70), *t*(19) = 3.18, *p* < 0.01, *d* = 1.08, whereas accuracy did not differ for eyebrows and eyes (*d’* eyebrows = 1.86, SD = 0.86 vs. *d’* eyes = 1.57, SD = 0.57), *t*(19) = 1.38, *p* = 0.18, *d* = 0.50, and for eyes and ears (*d’* eyes = 1.57, SD = 0.57 vs. *d’* ears = 1.01, SD = 0.70), *t*(19) = 2.39, *p* = 0.03, *d* = 0.77. The interaction of feature and instruction was not significant, *F*(2,57) = 0.33, *p* = 0.72, *ƞ*_*p*_^*2*^ = 0.01.

An analogous ANOVA of *criterion* did not show a main effect of feature, *F*(2,57) = 0.96, *p* = 0.39, *ƞ*_*p*_^*2*^ = 0.03, or instruction, *F*(1,57) = 3.24, *p* = 0.08, *ƞ*_*p*_^*2*^ = 0.05, but revealed an interaction between these factors, *F*(2,57) = 7.94, *p* < 0.001, *ƞ*_*p*_^*2*^ = 0.22. A series of paired-sample *t*-tests (with alpha adjusted to 0.05/3 = 0.017 for three comparisons) revealed a shift from a mismatch to a match bias from before to after instruction in the eyes condition (*criterion* before = 0.39, SD = 0.54 vs. *criterion* after = -0.13, SD = 0.52), *t*(19) = 4.61, *p* < 0.001, *d* = 0.42, whereas *criterion* before and after instructions did not differ in the eyebrows condition (*criterion* before = 0.23, SD = 0.75 vs. *criterion* after = 0.29, SD = 0.39), *t*(19) = 0.46, *p* = 0.65, *d* = 0.10, and the ears condition (*criterion* before = 0.03, SD = 0.48 vs. *criterion* after = 0.11, SD = 0.43), *t*(19) = 0.72, *p* = 0.48, *d* = 0.17.

#### Response times

**Fig 2:**
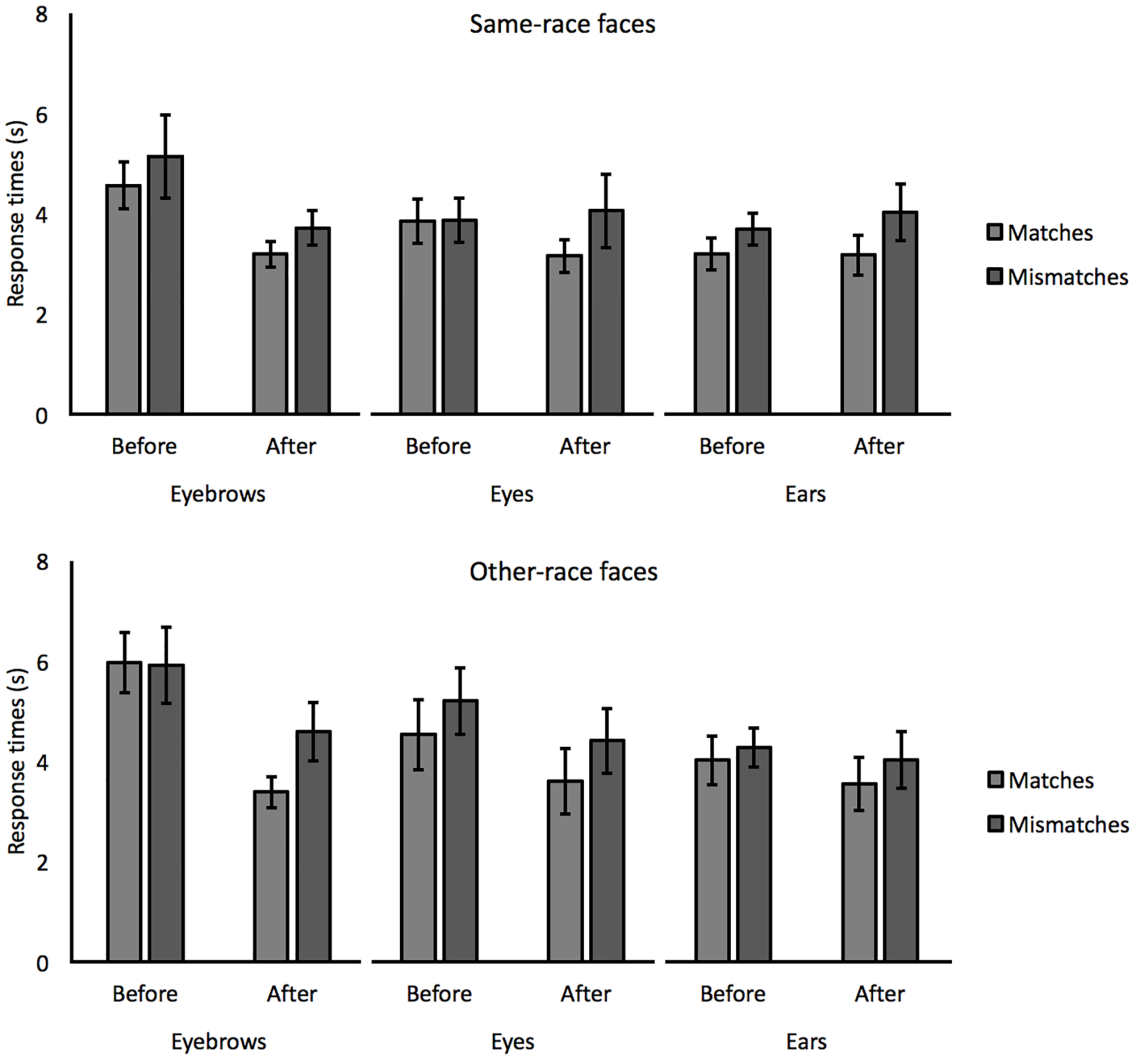
Response times for match and mismatch trials for same-race and other-race faces before and after the administration of eyebrows, eyes and ears feature instructions in Experiment 1. Error bars reflect standard errors of the means.

Although task instructions emphasized accuracy, response times were also analyzed to explore whether the improvement with instruction in the eyebrows condition might reflect a speed-accuracy trade-off. For this analysis, the mean response times were calculated for correct responses and are illustrated in Fig 2. For match pairs of same-race (Arab) faces, a 3 (feature) x 2 (instruction) mixed-factor ANOVA revealed a main effect of instruction, *F*(1,57) = 7.78, *p* < 0.01, *ƞ*_*p*_^*2*^ = 0.12, due to faster response times after instruction (3.87 vs. 3.17 seconds), but a main effect of feature, *F*(2,57) = 1.25, *p* = 0.29, *ƞ*_*p*_^*2*^ = 0.04, and an interaction were not found, *F*(2,57) = 2.46, *p* = 0.10, *ƞ*_*p*_^*2*^ = 0.08. A 3 (feature) x 2 (instruction) ANOVA for response times to mismatches did not show main effects of instruction, *F*(1,57) = 0.89, *p* = 0.35, *ƞ*_*p*_^*2*^ = 0.02, or feature, *F*(2,57) = 0.36, *p* = 0.70, *ƞ*_*p*_^*2*^ = 0.01, but a marginally significant interaction between these factors, *F*(2,57) = 3.16, *p* = 0.05, *ƞ*_*p*_^*2*^ = 0.10. A series of paired-sample *t*-tests (with alpha adjusted to 0.05/3 = 0.017 for three comparisons) showed a trend for faster response times after instructions in the eyebrows condition (5.14 vs. 3.72 seconds), *t*(19) = 2.30, *p* = 0.03, *d* = 0.49, but not in the eyes condition (3.87 vs. 4.06 seconds), *t*(19) = 0.38, *p* = 0.71, *d* = 0.07, or in the ears condition (3.69 vs. 4.03 seconds), *t*(19) = 0.64, *p* = 0.53, *d* = 0.15. Thus, these data indicate that the improvement in the eyebrow instruction condition does not reflect a speed-accuracy trade-off.

The same analysis was also performed for other-race (Caucasian) faces. A 3 (feature) x 2 (instruction) ANOVA for response times to matches revealed a main effect of instruction, *F*(1,57) = 12.03, *p* < 0.001, *ƞ*_*p*_^*2*^ = 0.17, due to faster response times after instruction (4.84 vs. 3.52 seconds), but a main effect of features, *F*(2,57) = 1.01, *p* = 0.37, *ƞ*_*p*_^*2*^ = 0.03, and an interaction between these factors was not found, *F*(2,57) = 2.82, *p* = 0.07, *ƞ*_*p*_^*2*^ = 0.09. Similarly, a 3 (feature) x 2 (instruction) ANOVA for mismatches revealed a main effect of instruction, *F*(1,57) = 6.73, *p* < 0.05, *ƞ*_*p*_^*2*^ = 0.11, due to faster response times after instruction (5.14 vs. 4.34 seconds), but not a main effect of features, *F*(2,57) = 1.02, *p* = 0.37, *ƞ*_*p*_^*2*^ = 0.03, or an interaction, *F*(2,57) = 1.06, *p* = 0.35, *ƞ*_*p*_^*2*^ = 0.04.

### Discussion

This experiment demonstrates that instruction to focus on specific facial features enhances face-matching accuracy. However, this advantage was not universally found. For the three features under investigation here, this enhancement was observed after instruction to focus on the eyebrows, but no such effect was present for the eyes, and a decline in accuracy was observed in the ears condition. One way to reconcile these findings could be that not all features are equally informative in face matching. Drawing attention to a feature that is less beneficial for identification (such as the ears in this case) may limit attention to more informative features (such as eyebrows), leading to an actual reduction in accuracy. We also note that the enhancement in accuracy that was observed in the eyebrows condition here was evident in overall accuracy, though a breakdown of the data indicates that this effect is carried primarily by performance on identity match trials.

These results converge with a recent investigation, which found that similarity ratings of facial features prior to matching decisions also enhance accuracy [29]. As in our study, this effect was most pronounced for identity matches. Moreover, similarly to performance with identity mismatches in the current experiment, Towler et al. [29] observed a consistent, though non-significant, reduction in performance for such face pairs. However, in Towler et al.’s [29] study, the diagnosticity of similarity ratings for matching decisions was greater for ears than eyes and foreheads. By contrast, similarity ratings for eyebrows were not examined in that study, whereas only instruction to attend to this specific feature conferred an improvement in the experiment reported here.

At present, it is not clear what drives these specific differences. One possibility is that some of these disparities, such as the effects with ears, might reflect differences in stimulus treatment. In the current experiment, the face stimuli were cropped, which may have altered the outline of the ears and contributed to a reduction in accuracy in the ear-instruction condition (see Fig 1). By contrast, Towler et al. [29] employed uncropped faces, thus preserving ear shape. A related explanation could be that these disparities reflect different characteristics of the underlying stimulus sets. The most diagnostic features for making an identification vary across faces [23,45]. Consequently, the features that are *generally* most useful for making matching decisions might also vary across stimulus sets. One might therefore expect that different features will be beneficial for enhancing accuracy for different stimuli.

Our study provides tentative support for this suggestion, by showing that eyebrow feature instructions enhanced accuracy for same-race but not for other-race faces. There is evidence that different facial features, such as the nose and eye regions, carry identity information in African and Caucasian faces, and that instruction to focus on race-specific individuating features can help to increase recognition accuracy [41,42]. If the same- and other-race faces that were employed here also possessed specific individuating features, then that could explain why feature instructions enhanced accuracy for one stimulus set but not the other. We note, however, that Towler et al. [29] also found that their accuracy improvements did not generalize to another stimulus set of same-race (Caucasian) faces. Thus, the absence of a feature instruction improvement for other-race (Caucasian) faces in the current study might not reflect race *per se*, but could represent a broader limitation in generalization across face sets.

These considerations raise the question of how robust the current effect is. We therefore conducted a second experiment to determine whether the eyebrow instruction advantage replicates with a different set of same-race stimuli. In addition, Experiment 1 also assessed face matching only under highly optimized conditions, by comparing same-day photographs of faces. Such stimuli are employed typically in laboratory tasks to establish best-possible performance but fail to capture the within-person variation that is encountered in realistic settings, such as the differences in a person’s appearance over days and months [11]. In Experiment 2, we therefore also sought to examine whether the eyebrow feature instruction effect persists under conditions that require matching of different-day face images.

## Experiment 2

Experiment 2 sought to replicate the eyebrow instructions improvement that was observed in Experiment 1 with a new set of same-race stimuli to determine the robustness of this effect. Observers were now asked to match pairs of faces that comprised same-day photographs or different-day photographs that were taken months apart. If the effect observed in Experiment 1 is robust, then it should be replicated with the new set of same-day face matching stimuli. Moreover, if this effect persists under more realistic conditions, that capture greater within-person variability, then it should be found also with the different-day photographs. Due to the addition of this factor, this experiment focused on feature instructions for the eyebrows only.

### Method

#### Participants

Thirty-two undergraduate students from Qatar University volunteered to participate in this experiment (*M*_age_ = 19.7, *SD*_age_ = 1.2; 66% females). All reported normal or corrected to normal vision. None had participated in Experiment 1.

#### Stimuli

A total of 224 face pairs served as stimuli in this experiment. Half of these stimuli (112; 66 matches and 66 mismatches) were face pairs taken from an Arab face set that was highly similar in construction to the face matching pairs of Experiment 1. These stimuli were constructed from 84 identities, so that none of the identities occurred in more than one match pair and two mismatches across conditions. Thus, this stimulus set provided same-day face images for each target identity, which were presented in frontal view, under good lighting, with a neutral expression, and on a plain background (for a full description of this stimulus set, see [9]). The other half of the stimuli (112; 66 matches and 66 mismatches) were photos of the same models but taken several months later (mean delay = 17.2 months, SD = 7.3; for a full description, see [11]). In the stimulus pairs, all face photographs measured approximately 5 x 7 cm and were shown in greyscale. Example stimuli can be found in [11].

#### Procedure

As in Experiment 1, participants were tested individually in a laboratory on an Apple Macintosh laptop, which presented stimuli and recorded responses. The 224 face-matching pairs were divided into 4 counter-balanced sets of 56 trials. Each participant was given one of these face sets, which were broken down into two blocks of 28 face pairs. Each of these two blocks comprised seven same-day matches and mismatches, and seven different-day matches and mismatches comprising photograph pairs that were taken months apart. Across participants, the appearance of each stimulus was counterbalanced, so that each face pair was equally likely to appear in the experimental blocks before and after feature instructions.

In the experiment, these stimuli were displayed until a response was registered, and participants were asked to classify these as identity matches and mismatches as accurately as possible by pressing two labelled buttons on the computer keyboard. In between both blocks, observers were given feature comparison instructions emphasizing the importance of eyebrows for face matching accuracy. Different face pairs were shown in each block and presented in a unique random order. However, the presentation of each target face in an identity match or mismatch, and the presentation of the stimuli across blocks were counterbalanced across observers over the course of the experiment.

### Results

#### Accuracy

**Fig 3:**
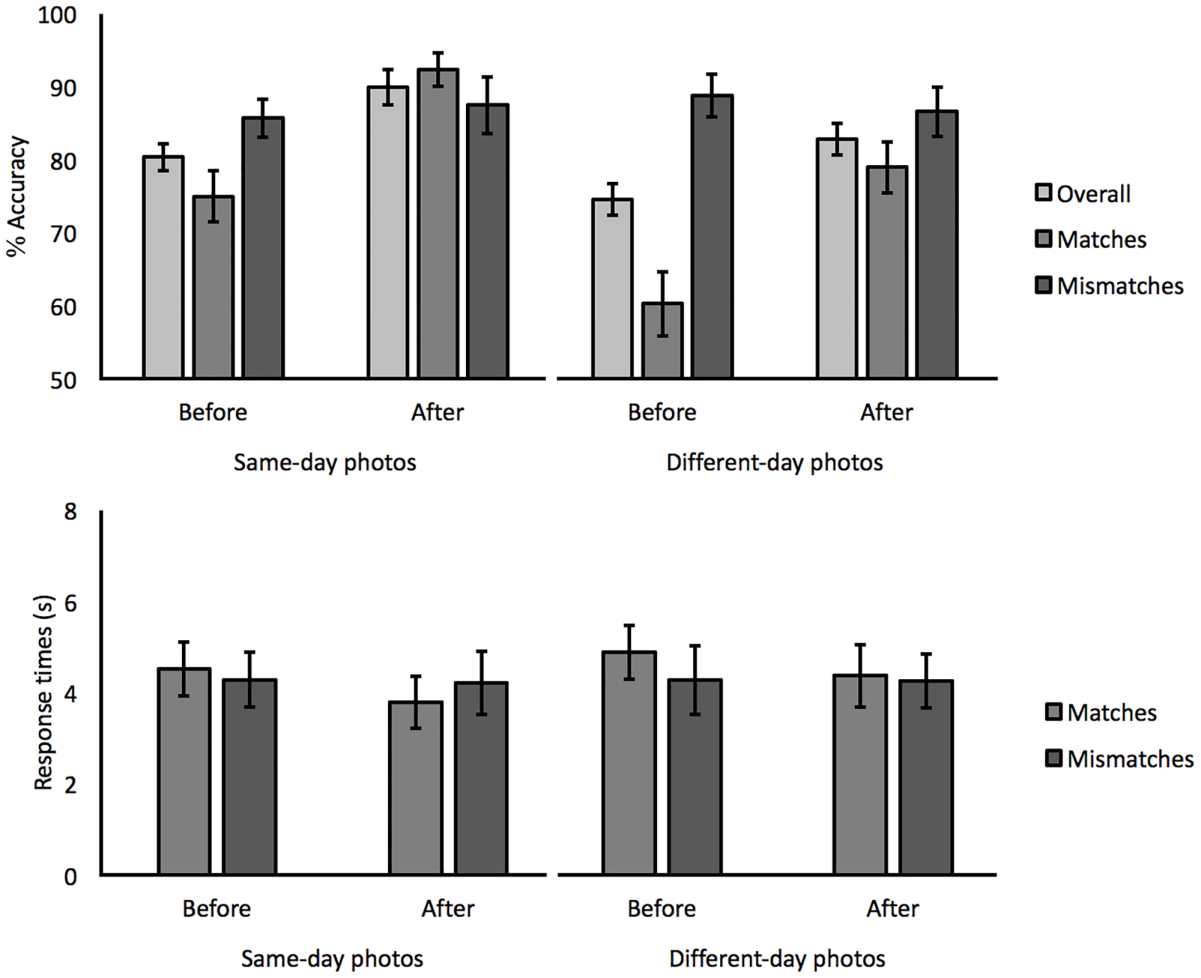
Overall, match and mismatch accuracy, and response times for same-day and different-day face pairs before and after the administration of eyebrows feature instructions in Experiment 2. Error bars reflect standard errors of the means.

As in Experiment 1, performance was analyzed in terms of the percentage accuracy of correct match and mismatch responses, as well as overall accuracy. These data are displayed in Fig 3. For overall accuracy, a 2 (face set: same-day vs. different-day) x 2 (instruction: before vs. after) within-subject ANOVA of overall accuracy revealed a main effect of face set, *F*(1,31) = 20.26, *p* < 0.001, *ƞ*_*p*_^*2*^ = 0.40, due to higher matching accuracy for face pairs that consisted of same-day (85.0%) than different-day (78.6%) photographs. In addition, a main effect of instruction was also found, *F*(1,31) = 15.74, *p* < 0.001, *ƞ*_*p*_^*2*^ = 0.34, reflecting higher matching accuracy after receiving feature instructions (86.4%) than beforehand (77.1%). The interaction of face set and instruction was not significant, *F*(1,31) = 0.28, *p* = 0.60, *ƞ*_*p*_^*2*^ = 0.01. An analogous ANOVA for match trials showed the same main effect of face set, *F*(1,31) = 52.69, *p* < 0.001, *ƞ*_*p*_^*2*^ = 0.63, with higher accuracy for same-day (84.3%) than different-day (69.6%) face pairs. A main effect of instruction was also found, *F*(1,31) = 41.82, *p* < 0.001, *ƞ*_*p*_^*2*^ = 0.57, due to higher accuracy after the feature instructions (85.7%) than beforehand (67.6%). Once again, the interaction of these factors was not significant, *F*(1,31) = 0.18, *p* = 0.68, *ƞ*_*p*_^*2*^ = 0.01. For mismatches, the main effects of face set, *F*(1,31) = 0.50, *p* = 0.48, *ƞ*_*p*_^*2*^ = 0.02, and instruction, *F*(1,31) = 0.01, *p* = 0.94, *ƞ*_*p*_^*2*^ = 0.00, and the interaction of these factors, *F*(1,31) = 1.34, *p* = 0.26, *ƞ*_*p*_^*2*^ = 0.04, were not significant.

#### Sensitivity and bias

The accuracy data were also transformed into signal detection measures of sensitivity (*d’*) and bias (*criterion*). For *d’*, a 2 (face set) x 2 (instruction) within-subject ANOVA showed a main effect of face set, *F*(1,31) = 9.93, *p* < 0.01, *ƞ*_*p*_^*2*^ = 0.23, and of instruction, *F*(1,31) = 18.65, *p* < 0.001, *ƞ*_*p*_^*2*^ = 0.38, and an interaction between these factors, *F*(1,31) = 4.38, *p* < 0.05, *ƞ*_*p*_^*2*^ = 0.12. A series of paired-sample *t*-tests (with alpha adjusted to 0.05/4 = 0.013 for four comparisons) showed that sensitivity was enhanced in the same-day condition after feature instruction compared to before these were administered (*d’* before = 2.09, SD = 0.77 vs. *d’* after = 3.30, SD = 1.25), *t*(31) = 4.62, *p* < 0.001, *d* = 1.16. A similar advantage was observed with different-day face pairs, though this did not survive adjustment for multiple comparisons (*d’* before = 1.88, SD = 0.83 vs. *d’* after = 2.51, SD = 1.07), *t*(31) = 2.53, *p* = 0.017, *d* = 0.66. In addition, sensitivity was comparable for same-day and different-day face pairs before instructions (*d’* same-day faces = 2.09, SD = 0.77 vs. *d’* different-day faces = 1.88, SD = 0.83), *t*(31) = 1.22, *p* = 0.23, *d* = 0.26, but was greater for same-day than different-day face pairs afterwards (*d’* same-day faces = 3.30, SD = 1.25 vs. *d’* different-day faces = 2.51, SD = 1.07), *t*(31) = 3.19, *p* < 0.01, *d* = 0.68.

For *criterion*, ANOVA revealed a main effect of face set, *F*(1,31) = 26.18, *p* < 0.001, *ƞ*_*p*_^*2*^ = 0.46, due to a greater bias to make mismatch responses for different-day face pairs (*criterion* same-day faces = 0.07, SD = 0.45 vs. *criterion* different-day faces = 0.40, SD = 0.55). A main effect of instruction was also found, *F*(1,31) = 16.67, *p* < 0.001, *ƞ*_*p*_^*2*^ = 0.35, due to a greater mismatch bias before than after receiving feature instructions (*criterion* before = 0.42, SD = 0.54 vs. *criterion* after = 0.06, SD = 0.52). The interaction of these factors was not significant, *F*(1,31) = 0.14, *p* = 0.71, *ƞ*_*p*_^*2*^ = 0.00.

#### Response times

Response times were analyzed to explore whether improvement with instruction might reflect a speed-accuracy trade-off. A 2 (face set) x 2 (instruction) within-subject ANOVA for correct match and mismatch responses yielded no main effects and interactions between factors, all *F*s (1,31) ≤ 2.88, *p*s ≥ 0.10, *ƞ*_*p*_^*2*^ ≥ 0.08 (see Fig 3).

### Discussion

This experiment confirms the improvement in face matching accuracy following instruction to focus on the eyebrows. Once again, this effect was observed in overall accuracy but was driven primarily by an improvement in performance on identity match trials. In addition to this replication, the current experiment extends these findings by showing that this effect was present during the matching of same-day face photographs as well as for different-day photographs, which were taken many months apart. The finding that the eyebrow instruction advantage is observed across different same-race stimulus sets, in Experiment 1 and Experiment 2, and across same-day and different-day photographs therefore points to a robust phenomenon.

## General discussion

This study examined whether the accuracy of face matching can be enhanced simply by verbal instruction to attend to specific facial features. In Experiment 1, feature instructions produced an advantage in accuracy when observers were asked to attend to the eyebrow regions of to-be-matched faces, which were depicted as optimized same-day photographs. In contrast, instruction to attend to eye regions did not affect performance, and instruction to attend to ears led to a decline in accuracy. We analyzed response times to explore whether this pattern might reflect a speed-accuracy trade-off in the eyebrows condition, which was not found to be the case. We therefore suggest that the eyebrows provided particular useful identity cues in the stimuli at hand here, whereas the ears were generally less useful for identification, such that attention to these regions resulted in a relative reduction in matching accuracy. Experiment 2 then replicated the improvement in face-matching accuracy with eyebrow instructions for a different set of same-day faces, and showed that this effect extends to different-day photographs of faces, which were taken months apart. Thus, a feature instruction advantage can be observed with highly-optimized same-day photographs for face matching, as well as with more ecologically valid different-day photographs that depict a person over longer time intervals.

These findings converge with a recent study, which showed that similarity ratings of facial features in face pairs influence subsequent identity-matching decisions, leading to an increase in accuracy [29]. Thus, both studies suggest that attention to features can enhance matching accuracy. Moreover, in both studies these effects appeared to be driven primarily by identity-match trials, further suggesting a common basis. However, these studies also differ in the features that appear to drive these effects. In Towler et al.’s [29] study, similarity ratings of the ears appeared to be most diagnostic for accurate identification decisions, whereas only instruction to attend to eyebrows led to an improvement in accuracy in the current study. A number of methodological differences exist between these studies that could explain these disparities. For example, the current study compared three facial features (eyebrows, eyes, ears) on a between-subject basis. In contrast, Towler et al. [29] asked participants to sequentially rate 11 different facial features, which did not include the eyebrows, prior to each identity-matching decision. Thus, these studies differ greatly in the type and number of features that observers were required to attend. Moreover, rating the similarity of *all* features *before* making a decision, as was the case in Towler et al.’s [29] study, and using a *specific* feature directly to *make* a decision, as was the case here, are different processes. Thus, it is also possible that how features are used determines which of these are most useful for enhancing face-matching decisions.

Finally, we note that both studies employed different stimulus sets. Thus, the possibility exists that the face stimuli in these sets may have differed in the defining features that are useful for making identification decisions, whereby the ears may have provided particularly clear identity information in Towler et al.’s [29] stimulus set and the eyebrows in the current study. This notion receives some support from the fact that these studies differed in terms of the race of the observers and the face stimuli, such that Towler et al. [29] utilized Caucasian faces whereas the current study was conducted with Arab observers and Arabic faces. A number of studies already suggest that faces of different races, such as African and Caucasian faces, carry different identity-defining features, and demonstrate that instruction to attend to specific individuating features increases recognition accuracy [41,42]. If Arab and Caucasian faces also differ systematically in such a way, then this might explain why different features are useful for enhancing matching accuracy in Towler et al.’s [29] and the current study.

To this point, it is noteworthy also that we compared the effect of feature instructions for same-race (Arab) with other-race (Caucasian) faces in Experiment 1. This revealed that feature instructions only enhanced accuracy for the same-race faces. This could reflect a facet of the other-race effect in face processing, whereby identification of other-race faces is not only more error-prone [34–36], but improvements in this ability are also disproportionately more difficult to elicit. However, it has also been shown that the other-race effect can be reversed when observers are cued to fixate specific facial features that observers from the other race would normally fixate upon [41,42]. Thus, the different findings with Arab and Caucasian faces in Experiment 1 might also reflect, again, that different facial features are informative for different races. Therefore, there could be a common basis also for explaining the different features that are informative for matching decisions in Towler et al.’s [29] and the current study, and the difference between same- and other-race faces here. On the other hand, we note that such an explanation might also predict that the same features that are most diagnostic of matching accuracy in Towler et al.’s [29] study are also useful for other-race faces here, on the basis that both tasks are based on Caucasian stimuli. However, Towler et al. [29] also failed to find generalization of the improvement in matching accuracy to another set of Caucasian faces. Thus, improvements in face matching accuracy might generalize poorly, irrespective of face race or of whether similarity ratings or feature instructions are employed for this purpose.

In conclusion, face matching is a difficult task, but one that is important for operational settings, such as passport control. Existing data indicate that methods for improving matching accuracy in such settings are required [1,10,15]. The current experiments suggest that the simple manipulation of instructing observers to attend to specific facial features *can* provide a route to improvement in this task. However, such a benefit was obtained only for identity matches, and we also found that attention to the ‘wrong’ features can reduce face-matching accuracy. Selection of features is therefore crucial for the application of this manipulation, but current data are still inconclusive about which features such approaches must generally focus on (c.f., [29]). We also failed to find generalization of improvement with feature instructions to other-race faces, indicating a further limitation of this approach. We suspect that these findings arise because distinguishing features might vary across different stimulus sets (see, e.g., [41,42]), across different identities within stimulus sets [23,45], and perhaps also for different images of the same person [18,46]. In that case, application of feature instructions would ultimately require an additional process to initially identify diagnostic features of individual faces. This might be achieved in future studies by asking participants to rate individual features [43], or by exploring perception of identity when different features are occluded systematically [32]. As we only possessed suitable stimuli of male Arab faces for the current experiments, future studies should also extend to female faces, and clarify whether the absence of generalization across faces from different races here reflects observer or stimulus characteristics.

## Supporting information

S1 Data S1 Data for the experiments reported in this study. (RAR)
